# A Sensitive, Reproducible and Objective Immunofluorescence Analysis Method of Dystrophin in Individual Fibers in Samples from Patients with Duchenne Muscular Dystrophy

**DOI:** 10.1371/journal.pone.0107494

**Published:** 2014-09-22

**Authors:** Chantal Beekman, Jessica A. Sipkens, Janwillem Testerink, Stavros Giannakopoulos, Dyonne Kreuger, Judith C. van Deutekom, Giles V. Campion, Sjef J. de Kimpe, Afrodite Lourbakos

**Affiliations:** Prosensa Therapeutics BV, Leiden, the Netherlands; University of Minnesota, United States of America

## Abstract

Duchenne muscular dystrophy (DMD) is characterized by the absence or reduced levels of dystrophin expression on the inner surface of the sarcolemmal membrane of muscle fibers. Clinical development of therapeutic approaches aiming to increase dystrophin levels requires sensitive and reproducible measurement of differences in dystrophin expression in muscle biopsies of treated patients with DMD. This, however, poses a technical challenge due to intra- and inter-donor variance in the occurrence of revertant fibers and low trace dystrophin expression throughout the biopsies. We have developed an immunofluorescence and semi-automated image analysis method that measures the sarcolemmal dystrophin intensity per individual fiber for the entire fiber population in a muscle biopsy. Cross-sections of muscle co-stained for dystrophin and spectrin have been imaged by confocal microscopy, and image analysis was performed using Definiens software. Dystrophin intensity has been measured in the sarcolemmal mask of spectrin for each individual muscle fiber and multiple membrane intensity parameters (mean, maximum, quantiles per fiber) were calculated. A histogram can depict the distribution of dystrophin intensities for the fiber population in the biopsy. This method was tested by measuring dystrophin in DMD, Becker muscular dystrophy, and healthy muscle samples. Analysis of duplicate or quadruplicate sections of DMD biopsies on the same or multiple days, by different operators, or using different antibodies, was shown to be objective and reproducible (inter-assay precision, CV 2–17% and intra-assay precision, CV 2–10%). Moreover, the method was sufficiently sensitive to detect consistently small differences in dystrophin between two biopsies from a patient with DMD before and after treatment with an investigational compound.

## Introduction

Duchenne muscular dystrophy (DMD) is characterized by progressive muscle fiber degeneration as a result of a dystrophin deficiency at the muscle fiber sarcolemmal membranes. The underlying mutations in the *DMD* gene are typically deletions of one or more exons (in 63% of patients) interrupting the open-reading frame. RNA-modulating therapy is the most advanced and promising strategy for this severe childhood muscle disease [1]. Several antisense oligonucleotides (AONs) are currently in (pre)clinical development. AON-induced exon skipping can restore the open-reading frame and, although the deletion and additional removal of an extra (flanking) exon seems paradoxical, the protein itself clearly allows such modification, as demonstrated in patients with the typically milder phenotype, Becker muscular dystrophy (BMD). Mutations in these patients result in an internally shortened but in-frame transcript and a dystrophin protein that is functional, especially when the mutation is located in the central rod domain between exons 10 and 60.

In current clinical studies aiming to increase dystrophin expression in patients with DMD, the pharmacodynamic effect of treatment can be assessed at the RNA or protein level. Such data can provide support for the mechanism of action and dose selection. Dystrophin bioanalytical assays need to consider “trace” dystrophin expression and “revertant” fibers. The expression of very low levels of trace dystrophin in patients with DMD has been observed before [1], [2], but emerging methodologies on imaging and quantification of expression levels have shown that trace dystrophin is expressed more commonly in nearly all fibers in the majority of DMD muscle biopsies [3]–[7]. The incidence of revertant fibers that express relatively high levels of dystrophin is generally low (<5%) and appears to be dependent on the deletion and can reach 14% [6], [7]. Trace dystrophin and revertant fibers can be recognized and accounted for in immunofluorescence analysis (IFA) of muscle biopsy cross-sections. Similarly, the presence of fibrosis and adipose tissue in a biopsy can be identified and excluded from analysis of muscle biopsies in IFA. IFA has the advantage that it can detect the effect of treatment on dystrophin expression at the sarcolemma of the muscle fibers in a biopsy.

Historically, significant efforts have been made to objectively measure dystrophin intensities. One image analysis method determined the average dystrophin intensity from all fibers in an image that co-localized with a spectrin signal in a software-generated mask defining the sarcolemmal area [4]. Another method assessed dystrophin expression by manually placing circles on randomly selected areas of the sarcolemma of a selected number of fibers per biopsy and measured the maximum membrane intensity within that circle [5], [6]. It has been reported that the intensity of dystrophin may vary between fibers in a healthy control muscle [4], [5] and that this variability is even more pronounced in BMD and DMD muscle [2], [3], [5]. Therefore, it would be more informative to measure not only the average intensity of all sarcolemmal membranes in an image or the maximum intensity in a limited number of fibers, but to determine the complete dystrophin expression at the membrane of each individual fiber in a biopsy cross-section. Ideally, image analysis should be automated to allow for higher throughput and unbiased interpretation of expression in a fiber or an image.

Here, we present a standardized IFA method that is sensitive, operator-independent, and reproducible. It is based on imaging by confocal microscopy and semi-automated image analysis using computer software. Individual fibers are identified by spectrin co-staining and dystrophin intensity is measured at each fiber membrane to determine the distribution of dystrophin expression over individual fibers in the cross-section of a muscle biopsy. We demonstrate that the method is fit-for-purpose for the analysis and comparison of muscle samples from healthy controls and from patients with BMD and DMD.

## Materials and Methods

### Ethics Statement

Human control muscle tissues were obtained from a commercial tissue bank (Asterand, Hertfordshire, UK; http://asterand.com/Asterand/about/ethics.htm) and from the Vrije Universiteit Medical Center (VUMC, Amsterdam, the Netherlands). The use of post-mortem material was approved by the VUMC research committee (project 2011-67), where relatives have given explicit prior written consent that tissue taken at autopsy can be used for research, after completion of the diagnostic process and informed consents were approved by institutional review boards. BMD biopsies were kindly provided by the Leiden University Medical Center (LUMC, Leiden, the Netherlands) and approved by the LUMC medical ethics committee and after written informed consent was obtained from all subjects or legal representatives. DMD biopsies were obtained from patients participating in clinical studies (registered at clinicaltrials.gov; NCT01153932 and NCT01037309) after approval by the local medical ethics committees and after signed patient informed consent including the assessment of dystrophin. Please see our supporting information file for a full list of participating sites (Table S1).

### Muscle Biopsies

The size of the muscle biopsies varied but was typically around 5×5×5 mm. The muscle biopsies were frozen in 2-methylbutane cooled in liquid nitrogen as described previously [8]. Muscle biopsies from the tibialis anterior were obtained from patients diagnosed with DMD or BMD. BMD biopsies (BMD 1–4) were kindly provided by Prof. dr. J.J. Verschuuren (LUMC). DMD biopsies were obtained from patients participating in clinical studies. The specific DMD mutation is for each donor indicated in Table 1. Samples DMD 1–3, 5, and 6 were naïve biopsies before treatment. DMD subject 5 participated in clinical study PRO044-CLIN-01 (Clinicaltrials.gov NCT01037309) and biopsies were taken before and after treatment with five weekly injections of 12 mg/kg antisense oligonucleotide PRO044, an investigational compound that can induce exon skipping to correct the reading frame of the mRNA.

**Table 1:** Control, BMD, and DMD muscle samples used.

Donor	Dystrophin mutation
Control 1	N/A
Control 2	N/A
Control 3	N/A
BMD 1	Deletion exon 45–47
BMD 3	Deletion exon 45–47
BMD 4	Deletion exon 03–07
BMD 2	Ex.19:c.2380+3A>C
DMD 1	Deletion exon 48–50
DMD 2	Deletion exon 45–50
DMD 3	Deletion exon 50
DMD 4	Deletion exon 45
DMD 5	Deletion exon 45
DMD 6	Deletion exon 43

BMD, Becker muscular dystrophy; DMD, Duchenne muscular dystrophy; N/A, not applicable.

Muscle samples from control donors were obtained post-mortem from individuals not suffering from DMD and kindly provided by Prof. dr. H.W. Niessen (VUMC, control 1 and 3, following storage of the body under cooled conditions (<5°C) for a maximum of 12 hours) and Asterand (control 2, following storage of the body for 7 hours). From healthy control 1, samples from six different muscle groups were obtained: tibialis anterior, quadriceps, gastrocnemius, biceps, triceps, and soleus. From control 3, only a quadriceps sample was obtained and from control 2, only a tibialis anterior sample.

### Sectioning and Immunofluorescence Double-staining Procedure

Each experiment was performed by several operators and different operators were arbitrarily assigned to each of the subsequent processing steps (sectioning of muscle biopsies, staining of sections for dystrophin and spectrin, image acquisition by confocal microscopy, and software-assisted image analysis). Biopsy quality was assessed by hematoxylin and eosin staining and was routinely performed to evaluate freezing artefacts (biopsy handling, transport and storage) and relative amount of fibrotic and adipose tissue, and to ensure sufficient fibers for reliable IFA for dystrophin.

Using a cryotome (Thermo Fisher Scientific, Waltham, MA, USA), 8 µm muscle cross-sections were cut and placed on Superfrost Ultra Plus Microscope Slides (Thermo Fisher Scientific, VWR 631–0099). After drying at room temperature for 1 hour, the slides were placed in a −80°C freezer and used within 2 months. The staining procedure was performed at room temperature. Slides were removed from the freezer, air dried for 20 minutes, fixed with acetone for 1 minute, washed with PBS, blocked with PBS containing 0.05% Tween-20 and 5% horse serum for 1 hour, and subsequently washed with PBS. For each biopsy, two or four sections were stained. Double-staining for dystrophin and spectrin was performed with the following combination of antibodies: mouse monoclonal MANDYS106 rod-domain anti-dystrophin antibody [9], [10] combined with a rabbit anti-spectrin antibody, or rabbit polyclonal ab15277 C-terminal anti-dystrophin antibody combined with a mouse anti-spectrin antibody (Table 2). Cross-reactivity to utrophin has been a concern for C-terminal anti-dystrophin antibodies, but is highly unlikely for ab15277 as it is raised to a peptide epitope unique to dystrophin and is affinity purified. Sections were incubated for 2 hours with anti-dystrophin or isotype antibody alone followed by a 1-hour incubation with anti-dystrophin and anti-spectrin antibodies combined, or isotype and anti-spectrin antibody combined. After the incubation with the primary antibodies, the sections were washed with PBS and then incubated with the appropriate secondary antibodies combined for 1 hour (Table 2). After washing again with PBS, the slides were mounted with Vectashield Mounting medium (Vector Laboratories, Burlingame, CA, USA; Brunschwig 6-H-1400) and imaged on the same day (Table 2).

**Table 2:** Antibody reagents for immunofluorescence detection of dystrophin and spectrin.

Double-staining combination	Dystrophin primary antibody	Spectrin primary antibody	Secondary antibody antimouse	Secondary antibody antirabbit
MANDYS106 and spectrin	Mouse MANDYS106 [9] ^a^	Rabbit α-spectrin (Thermo Scientific PA1-46007)^c^	α-mouse AlexaFluor488 (Invitrogen A11029)^d^	α-rabbit AlexaFluor594 (Invitrogen A11037)^e^
Mouse isotype and spectrin	Mouse IgG2A (Sigma M5409)^b^	Rabbit α-spectrin (Thermo Scientific PA1-46007)^c^	α-mouse AlexaFluor488 (Invitrogen A11029)^d^	α-rabbit AlexaFluor594 (Invitrogen A11037)^e^
ab15277 and spectrin	Rabbit polyclonal (Abcam ab15277)^c^	Mouse α-spectrin (Novocastra NCL-SPEC1)^c^	α-mouse AlexaFluor594 (Invitrogen A11032)^e^	α-rabbit AlexaFluor488 (Invitrogen A11034)^d^
Rabbit isotype and spectrin	Rabbit polyclonal IgG (Abcam ab27478)^c^	Mouse α-spectrin (Novocastra NCL-SPEC1)^c^	α-mouse AlexaFluor594 (Invitrogen A11032)^e^	α-rabbit AlexaFluor488 (Invitrogen A11034)^d^

Dilution of primary antibodies in PBS containing 0.05% Tween, 5% FBS: ^a^1∶60,^ b^1∶300, ^c^1∶200.

Dilution of secondary antibodies in PBS containing 0.05% Tween: ^d^1∶250,^ e^1∶1000.

FBS, fetal bovine serum; Ig, immunoglobulin; PBS, phosphate-buffered saline.

### Image Acquisition Procedure by Confocal Microscopy

Images were obtained using a Zeiss LSM 710 confocal microscope to enhance sharp membrane images. The standard imaging process captures 3-µm thick Z-slices. During development, we observed that the results were not influenced when using multiple Z-slices from the 8-µm section. From each muscle cross-section, five non-overlapping images at 25× magnification were acquired. Areas of the section containing very few muscle fibers or extensive fibrosis and adipose cells or sectioning artefacts were avoided. For the corresponding isotype staining for each biopsy, three non-overlapping images were obtained per section.

Pinhole size, image resolution, color depth, scan speed, and averaging (number of scans per image) were the same for all experiments. When imaging for an experiment that included healthy control and BMD/DMD biopsies, the microscope settings for laser intensity and detector gain for the dystrophin and spectrin channels were adjusted using the healthy control sample (with the highest signal intensity) so that only a few saturated pixels were present in the acquired images for the healthy control. When imaging for an experiment aiming to compare DMD samples, the imaging parameters for laser intensity and detector gain for the dystrophin channel were optimized for fluorescent beads (InSpeck Green [505/515] Microscope Image Intensity Calibration Kit; Invitrogen I-14785; 0.3% beads; Carlsbad, CA, USA), using higher laser intensity (DMD settings). Higher laser intensity makes better use of the 12-bit dynamic range (4095 arbitrary units) to detect differences at low expression levels. The spectrin settings were again optimized for the sample with the highest spectrin intensity, usually one of the DMD samples. In a single experiment, all images (control, DMD, or BMD) are taken at the same microscope, laser, and detector settings.

### Image Analysis and Dystrophin Intensity Measurement Using Image Analysis Software

**Figure 1:**
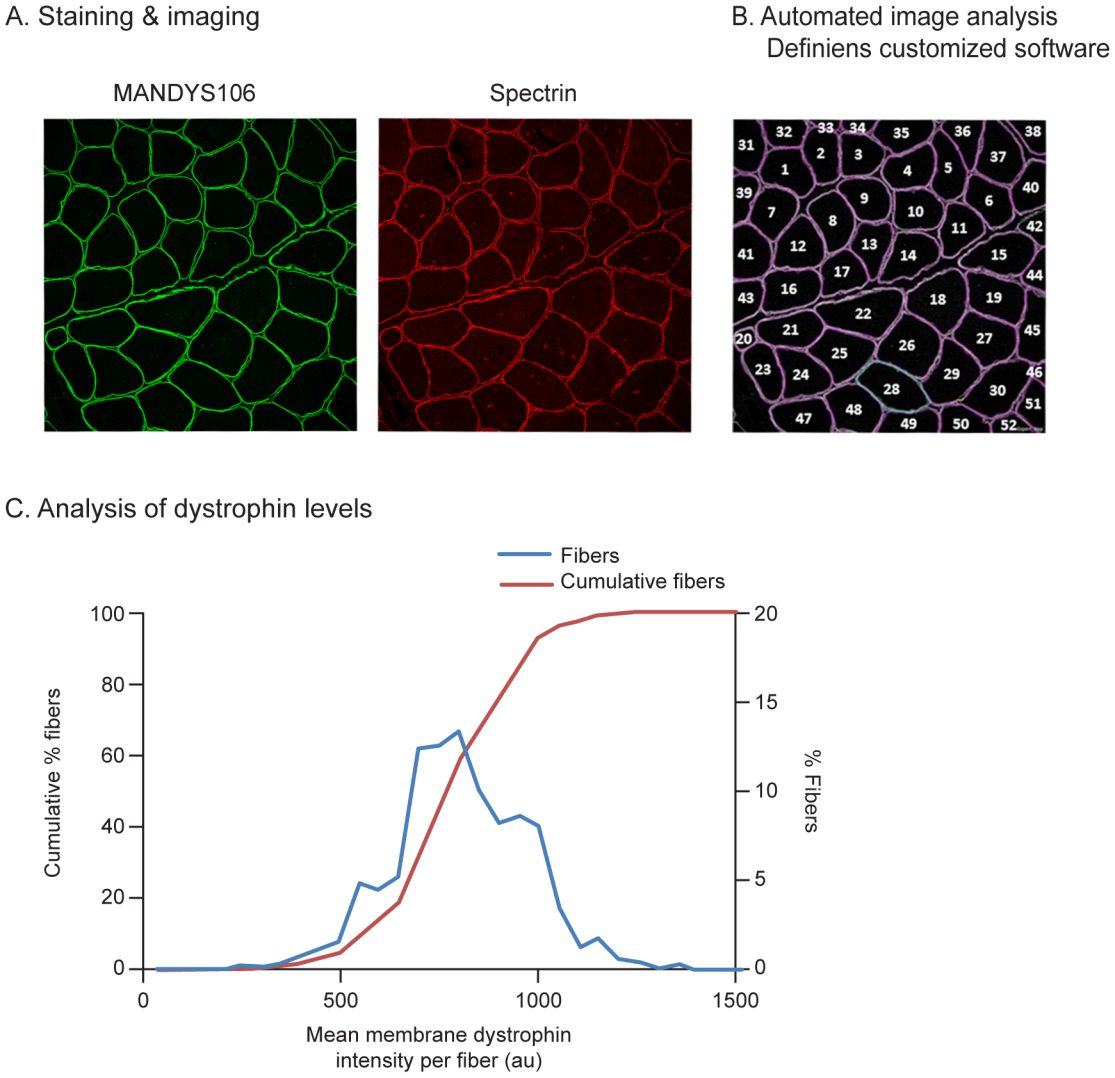
Immunofluorescence analysis of dystrophin intensities per fiber in a muscle fiber population. **A.** Cryosections of a healthy control muscle sample (control 3, quadriceps muscle) co-stained with anti-dystrophin antibody MANDYS106 and an anti-spectrin antibody. The sections were imaged using a confocal microscope, with the green channel (Alexa488) showing dystrophin and the red channel (Alexa594) showing spectrin. **B.** Images were analyzed using customized Definiens software, which automatically identifies individual muscle fiber membranes using their spectrin signal and measures the membrane dystrophin intensity per muscle fiber. **C.** Dystrophin intensity distribution in muscle fiber population analyzed, shown as a regular histogram (blue line) or a cumulative histogram (red line). (au: arbitrary units).

Image analysis was performed using Definiens Architect software (version 2.0) with a customized algorithm and application. Using the spectrin signal, the software identified the individual muscle fibers and determined within each fiber the sarcolemma and the cytoplasm. The software is able to distinguish between touching membranes of adjacent fibers using an algorithm based on maximum predicted membrane thickness and signal intensities from the individual fibers. On average, 400 fibers were analyzed per section (five images), for two or four sections per biopsy sample. Normally, at least 90% of the fibers in each image of a DMD patient-derived biopsy are sufficiently intact to be correctly identified (Figure 1B). The intensity of the dystrophin signal wass then determined. The dystrophin signal at the sarcolemma, as defined by spectrin, is considered to be dystrophin protein properly localized to the membrane. Images were processed automatically in batches, and identification of fibers was checked by operators. Mean intensity, minimum, maximum, and quantile dystrophin intensity values were determined per fiber. In addition, spectrin intensity per fiber was measured and morphology parameters calculated (such as cross sectional area of individual fibers and membrane thickness). The mean dystrophin membrane intensity (in arbitrary units [au]) is the average intensity of all pixels present in the spectrin surface area of an individual fiber. The quantile value of Q90 dystrophin intensity of a fiber, for instance, is the cut-off between 90% of pixels in the membrane with the lowest intensity and the 10% of pixels with the highest intensity. Generally, the “Q90–mean” of each fiber is used, which represents the mean intensity of the 10% of pixels with the highest intensity in the membrane of a fiber. The Q90-mean appears to represent what is observed by eye as the “brighter fluorescence” at the membrane and with high contrast to cytoplasm.

The dystrophin intensity differs per fiber. The distribution of the dystrophin levels in a fiber population from a muscle biopsy can be assessed and depicted as a histogram (Figure 1C). The average for the mean dystrophin level per fiber can be calculated for the entire fiber population, but the range for the dystrophin level per fiber can vary considerably within a biopsy cross-section. In healthy (non-DMD) control muscle, the mean dystrophin expression in a fiber may vary by threefold (in the range of 500–1350 au mean intensity per fiber). Alternatively, this histogram can also be displayed as a plot of cumulative percentage of fibers, which results in an S-curve (Figure 1C). The heterogeneity of dystrophin expression in the fibers determines the slope of the S-curve and a steeper slope depicts a more homogeneous dystrophin expression between fibers of a biopsy. An increase in overall dystrophin levels will make the cumulative dystrophin curve shift to the right.

### Statistical analysis

Reproducibility of the methodology has been assessed on the mean dystrophin intensity per fiber calculated for the biopsies.

Intra-assay precision: 2–4 replicate sections of a biopsy were analyzed in the same experiment and the percent coefficient of variation (CV) was calculated (CV%  =  standard deviation/average dystrophin intensity * 100% for the different sections of the biopsy).

Inter-assay precision was assessed qualitatively by ranking and quantitatively by calculation of the CV%. For ranking, the same biopsies were analyzed in different experiments and the dystrophin intensity measured was used to rank order the biopsies. The ranking of the biopsies between the different experiments was compared. For the CV%, the dystrophin intensity result of the same biopsy analyzed for different experiments on different days were used for the calculation (CV%  =  standard deviation/average dystrophin intensity * 100% for the different experiments on the biopsy).

To determine the statistical significance of the difference between pre- and post-treatment biopsy of subject DMD 4 analyzed in the same experiment, a linear mixed model was used. The dependent variable was log transformed in order to have a better normal distribution. Fixed term in the model was visit (before/after treatment) and random factors were sections within visits and images within sections.

## Results

### Method Optimization in Control Muscle Samples with Muscle and Donor Variability in Dystrophin Levels

**Figure 2:**
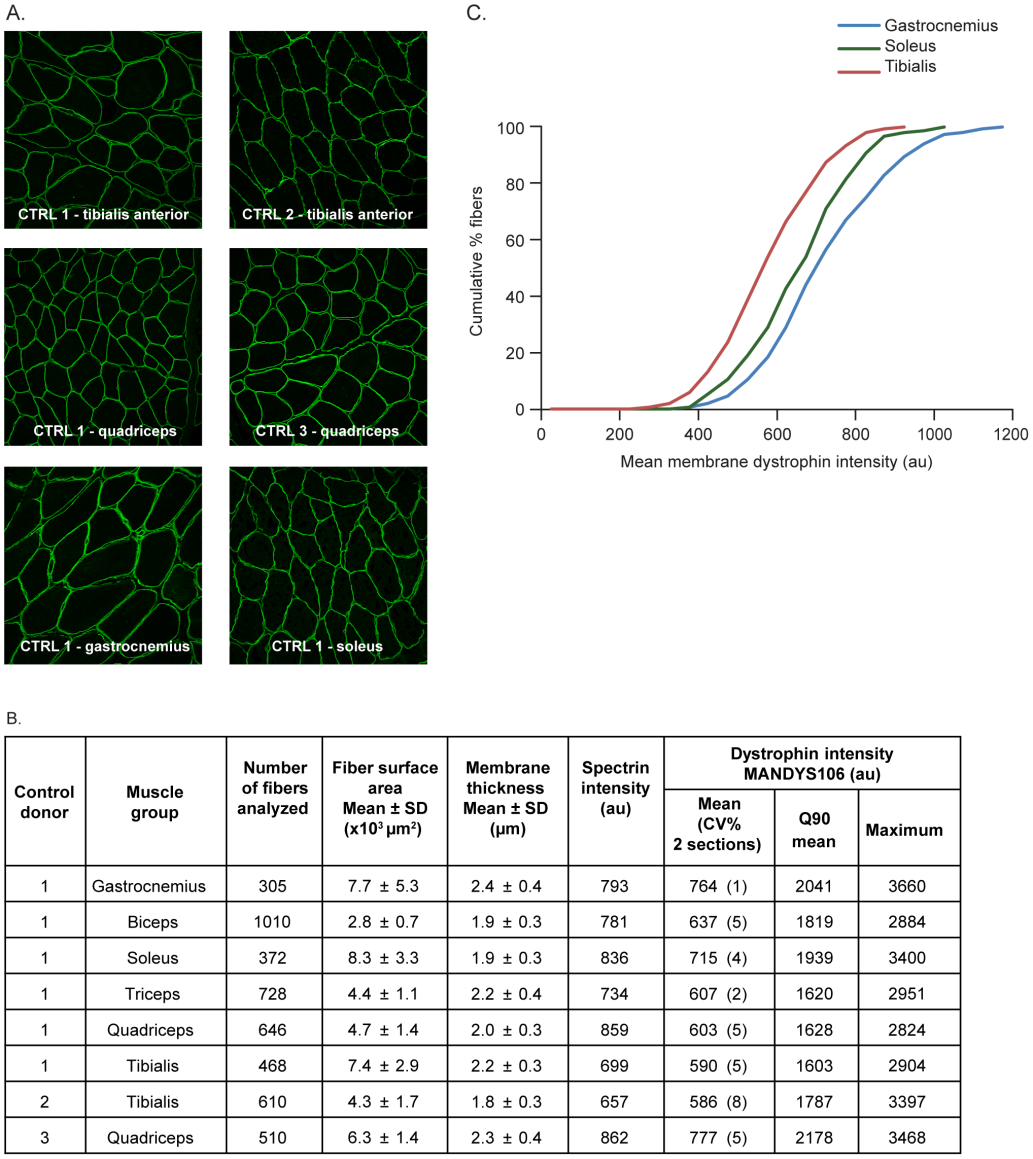
Dystrophin expression in healthy (non-DMD) control muscle samples from different muscle groups and different donors using MANDYS106. Eight muscle samples were compared within one experiment: six muscle groups from one donor (control 1) and two muscle groups from two other donors (controls 2 and 3). Analysis was performed on two sections per sample; imaging was performed with microscope settings suitable for imaging control samples (1% laser intensity). A. Representative immunofluorescence images of different muscle groups from Controls 1–3. B. Summary of numbers of fibers analyzed per samples, morphology parameters (fiber cross-section surface area and membrane thickness), mean spectrin intensities and dystrophin intensity parameters (mean, Q90 mean and maximum) of all the samples analyzed. C. Dystrophin intensity distribution in the fiber populations (cumulative plots) of three different muscle groups analyzed for control 1. (au: arbitrary units; CV%: coefficient of variation of replicate sections; CTRL: control; DMD: Duchenne muscular dystrophy; SD: standard deviation).

In a series of experiments, the staining procedures for anti-dystrophin MANDYS106 and ab15277 antibodies, and image capture and analysis using Definiens architect software, were optimized using healthy control muscle samples from different muscle groups and donors. The method showed that dystrophin intensities vary widely between individual fibers within one cross-section and, therefore, comparison of samples should consider the distribution of membrane dystrophin intensities over individual fibers in a sample. In a representative experiment with MANDYS106 (Figure 2A), samples from six different muscle groups of one donor (control 1) were analyzed. The mean dystrophin membrane intensity and Q90 mean values per fiber varied between muscle groups. The highest dystrophin intensities were measured in the gastrocnemius muscle for the mean dystrophin per fiber (average  = 764 au per fiber) and also for the mean of the 10% brightest pixels (average of the Q90-mean  = 2041 au). The lowest dystrophin intensities were measured in the tibialis anterior muscle (mean of 590 au and Q90-mean of 1603 au per fiber) (Figure 2B). The distribution of dystrophin intensities in the entire fiber population differed between muscle groups (Figure 2C). The fiber dystrophin intensities in the tibialis anterior and quadriceps muscles were also analyzed for two different donors (control 2 and 3, respectively), and varied by up to 29% (quadriceps) between donors. The observation that dystrophin intensity varies between fibers within a healthy control muscle has been previously reported using epifluorescence and confocal microscopy [4]–[6]. Although the variance in dystrophin intensities between muscle groups and between donors, observed reproducibly in independent experiments using different cross-sections, is suggestive for differential dystrophin expression in muscle groups and donors, sample variation (site and timing of sampling) should also be taken into account.

### Detection Range and Reproducibility in Control, BMD, and DMD Muscle Samples

**Figure 3:**
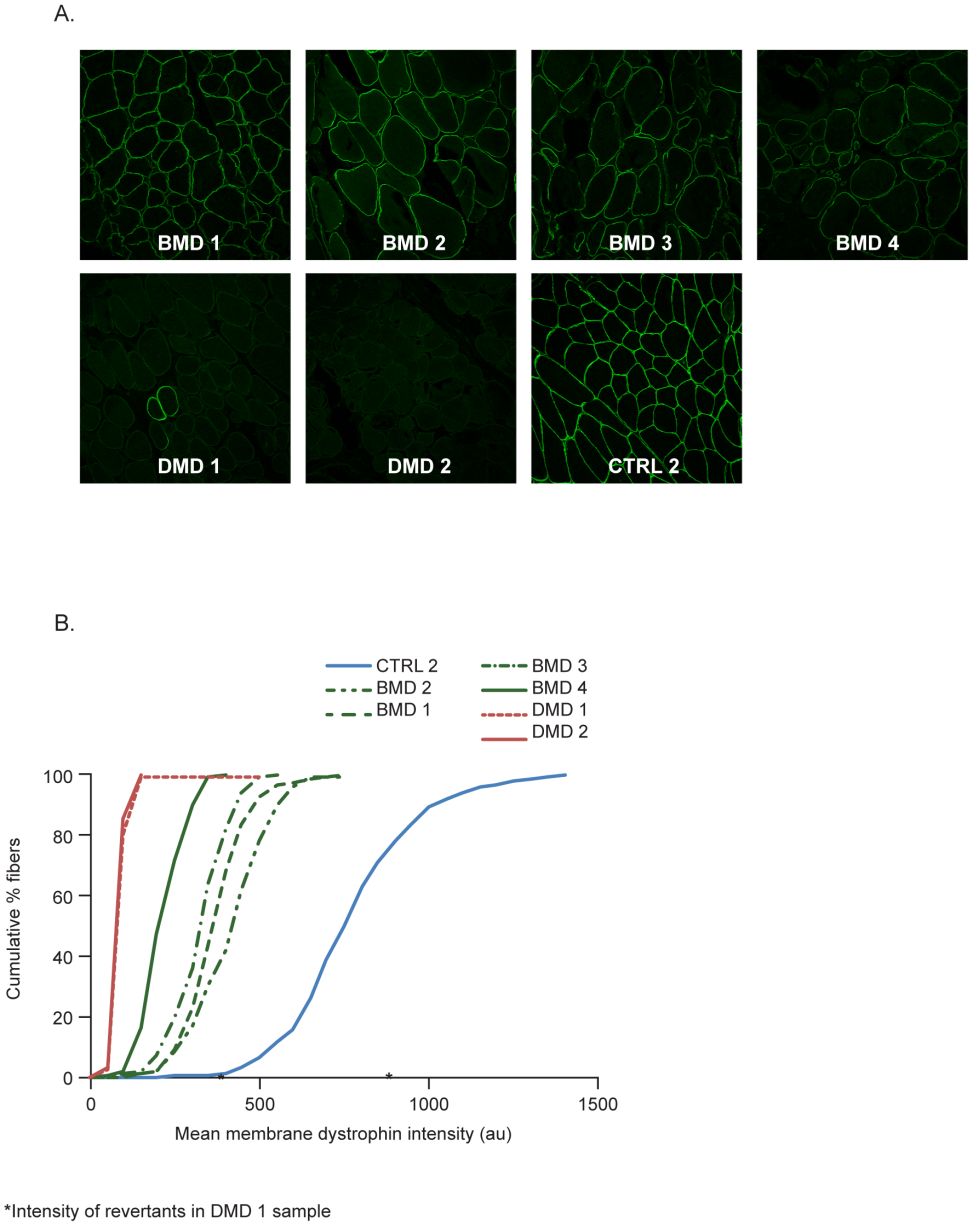
Dystrophin intensity in control muscle samples, and pre-treatment DMD and BMD muscle biopsies. **A.** Representative immunofluorescence images (1 of 10 images) from the tibialis anterior muscle samples (from one control subject (CTRL2), two patients with DMD and four patients with BMD. Analysis was performed in the same experiment using the MANDYS106 antibody and ‘control’ imaging settings (1% laser intensity). **B.** Corresponding dystrophin membrane intensity distribution in the fiber populations analyzed (cumulative graphs). (au: arbitrary units; BMD: Becker muscular dystrophy; CTRL: control; DMD: Duchenne muscular dystrophy).

The detection range and reproducibility of the method was assessed for two different anti-dystrophin antibodies, MANDYS106 and ab15277. Biopsies from the tibialis anterior muscle of donors with DMD (n = 2) or BMD (n = 4) were analyzed and compared to control 2. In a representative experiment (Figure 3A), MANDYS106 staining showed that the average mean dystrophin intensity per fiber was very low in the DMD biopsies (approximately 82 au) and intermediate in the BMD samples (ranging from 213 to 367 au) when compared to the 768 au in the control 2 sample (Figure 3B, Table 3). The intra-assay precision (CV%) ranged between 3 and 13% for replicate sections. The cumulative histogram plots of the control and BMD samples again highlight the typical variability in dystrophin expression between individual fibers in one cross-section but also show that the differences between patient biopsies apply across all fibers (Figure 3B). In addition, revertant fibers in the DMD samples had a higher dystrophin intensity, which was in the range of control muscle intensity (indicated by the asterisks in Figure 3B).

**Table 3:** Reproducibility of detecting differences in dystrophin expression in muscle biopsies with a wide range of dystrophin levels from healthy controls to BMD and DMD samples by immunofluorescence using MANDYS106 antibody and Definiens intensity measurements (at ‘control’ microscope settings).

Sample	Experiment	Number of sections	Number of fibers analyzed	Cross sectional area of individual fibers; Mean ± SD (x10^3^ µm^2^)	Dystrophin membrane intensity MANDYS106 (au)					
					Mean	Intra-assay precision CV% sections	Inter-assay precision CV% experiments	Q90 mean	Maximum	Ranking
Control 2	1	2	625	4.4±1.8	929	13%	13%	2397	3609	1
	2	2	544	4.3±1.9	768	3%		2169	3640	1
BMD 1	1	2	262	4.8±2.7	415	3%	9%	1224	2425	2
	2	2	337	4.9±2.2	367	6%		1076	2195	3
BMD 3	1	2	173	7.2±3.6	387	13%	4%	1125	2366	3
	2	2	221	7.2±3.8	412	7%		1195	2439	2
BMD 4	1	2	250	5.8±3.9	359	4%	7%	1062	2152	4
	2	2	102	7.7±4.9	323	6%		993	2122	4
BMD 2	1	2	300	4.4±3.3	200	7%	4%	668	1553	5
	2	2	197	6.4±4.2	213	4%		689	1552	5
DMD 1	1	2	1102	2.3±1.1	58	6%	24%	154	341	6
	2	2	739	3.0±1.8	82	13%		198	399	6
DMD 2	1	2	937	2.6±1.5	44	10%	43%	115	243	7
	2	2	816	2.5±1.4	82	6%		184	334	7

The intra-assay precision (CV%) was calculated for the mean dystrophin intensity of multiple sections of a biopsy/sample analyzed in the same experiment. Inter-assay precision between experiments performed on different days was assessed in two ways by ranking of the samples based on the dystrophin intensity in each experiment and by calculating the CV% from the mean dystrophin intensity in each experiment (experiment 1 and 2 performed 102 days apart).

BMD, Becker muscular dystrophy; CV, coefficient of variation; DMD, Duchenne muscular dystrophy; SD, standard deviation.

To evaluate the inter-assay precision of the method, two experiments were performed on different days with different operators for muscle biopsy staining, image acquisition, and processing. Muscle samples were ranked (1–7) based on their average dystrophin intensity. The ranking in the two experiments was very consistent (Table 3), except for two BMD samples (BMD 1 and 3) that expressed very similar dystrophin levels (7% and 11% difference between the 2 samples in 2 experiments). Using the dystrophin intensity arbitrary units measured for the same samples between experiments, the CV% was calculated to range between 4 and 13% for the control and BMD samples and between 24 and 43% for the DMD biopsies.

The consistency of dystrophin expression was also evaluated using the same control, BMD, and DMD samples but with staining with ab15277, a polyclonal rabbit antibody directed to the C-terminal end of the protein. Using ab15277, the lowest dystrophin intensity was measured in the DMD biopsies and higher levels in the BMD and control samples. The ab15277 antibody appears to be highly specific for dystrophin detection as indicated by the ability to detect also trace dystrophin and revertant fibers (Figure S1) and the very low staining observed in DMD biopsies is in agreement with previous work [4], [11]. The same ranking of samples (1–7) was observed for two ab15277 experiments, and the MANDYS106 staining (Figure S1B). These results indicate that the IFA method using two different anti-dystrophin antibodies can reproducibly detect a wide range of dystrophin intensity levels. The resulting dystrophin intensity distribution plots are highly informative and reproducibly ranked the different DMD and BMD muscle biopsies in an operator-independent manner.

Noteworthy are the mean spectrin intensities measured per fiber, which were typically higher in the DMD and BMD samples compared with the control sample (Figure S2). This was observed throughout all experiments (data not shown) and is in agreement with earlier reports describing qualitative assessments of immunofluorescence images for spectrin in fibers in diseased tissue [12], [13].

### Baseline Trace Dystrophin Intensities in DMD Muscle Biopsies

The inter-assay precision is lower for trace dystrophin levels in DMD biopsies and these can become indistinguishable at 1% laser intensity (Figure 3). To optimize imaging settings in this low range of detection, a 7% confocal laser intensity was used to be able to measure such subtle differences reproducibly. Fluorescent beads are used to determine the settings for the confocal microscopy and image acquisition and can also be used as a reference value between experiments. The number of fibers analyzed in a DMD muscle cross-section varied depending on the size of the muscle fibers and/or the disease state (i.e. the presence of connective and adipose tissue). Generally, each image contained an average of 80 fibers. With two sections analyzed per biopsy, between approximately 400 and 1350 fibers were included (average 800 fibers). With four sections per biopsy a maximum of ∼3000 fibers was analyzed.

**Figure 4:**
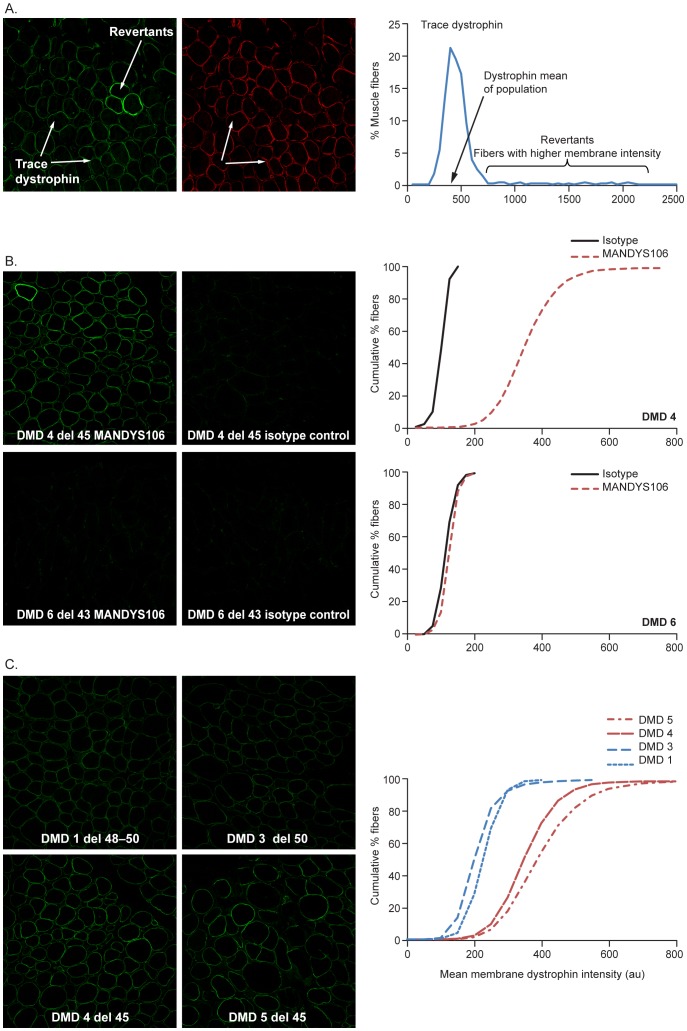
Dystrophin signal specificity using MANDYS106. Immunofluorescence intensities measured in pre-treatment biopsies of different patients with DMD at microscope settings suitable for imaging DMD samples (7% laser intensity). **A.** (Left): Dystrophin staining (AlexaFluor488). All fibers exhibit varying intensities of dystrophin (trace dystrophin) and certain fibers termed “revertants” express higher levels of dystrophin. (Middle): Spectrin staining (AlexaFluor594): Fibers with equivalent spectrin intensities exhibited varying levels of dystrophin staining. (Right): Histogram of the mean dystrophin intensity in the fiber population depicting the fibers expressing trace dystrophin and revertant fibers. The mean dystrophin intensity of the fiber population expressing trace dystrophin is indicated by the arrow. **B.** Specificity of MANDYS106 antibody compared with isotype control for measuring dystrophin expression. Immunofluorescence images of MANDYS106 (left) or negative control isotype staining (right) of patients DMD 4 (deletion exon 45) and DMD 6 (deletion exon 43), and the corresponding histograms of mean dystrophin intensity (cumulative graphs). **C.** Dystrophin intensities in patients with DMD with different exon deletions (DMD patient 1: deletion exon 48–50; DMD patient 3: deletion exon 50; DMD patient 5: deletion exon 45; DMD patient 6: deletion exon 45). These samples were analyzed in the same experiment. (au: arbitrary units; del: deletion; DMD: Duchenne muscular dystrophy).

Using these optimized settings (7% laser intensity), variation in dystrophin expression per fiber within a DMD biopsy and between DMD biopsies also become apparent (Figure 4A). Whereas the dystrophin intensities vary between fibers, the spectrin intensities are generally similar and no correlation with the variability in dystrophin detection was observed. The intensity of trace dystrophin varied for individual fibers in a muscle biopsy population, typically between 200 and 800 au (eg, DMD example, Figure 4). For comparison, the intensity of the revertant fibers ranged from 800 to 2500 au.

The specificity of the detection of trace dystrophin in these DMD biopsies was confirmed by the increased signal intensity for the anti-dystrophin MANDYS106 antibody compared to the isotype control antibody control for aspecific binding (mouse IgG2a). Isotype staining for background is routinely applied in each experiment for each biopsy. The specificity of trace dystrophin was also confirmed by the DMD 6 biopsy, collected from a patient with DMD with a deletion of exon 43. As the epitope for the MANDYS106 antibody is encoded by this exon, no trace dystrophin expression was detected in this biopsy (Figure 4B). In contrast, in sample DMD 4 (deletion of exon 45), MANDYS106 staining exceeded isotype control staining (Figure 4B).

Trace dystrophin expression is typically much higher in patients with exon 44 flanking deletions (DMD 4 and 5) than in patients with exon 51 flanking deletions (DMD 1 and 3) (Figure 4C, Table 4). The cumulative distribution plots of the membrane dystrophin intensity per fiber show a clear shift of the whole fiber population to higher dystrophin intensities for exon 44 samples DMD 4 and DMD 5 compared with exon 51 samples DMD 1 and DMD 3. This is in accordance with the higher levels of spontaneous exon 44 skipping on the RNA level that we detected in multiple non-treated muscle cell cultures and baseline biopsies of patients with exon 44 flanking deletions (data not shown).

**Table 4:** Reproducibility of detecting differences in dystrophin expression in DMD muscle biopsies by immunofluorescence using MANDYS106 antibody and Definiens intensity measurements (at high laser settings).

Sample	Experiment	Day	Number of sections	Number of fibers	Dystrophin membrane intensity MANDYS106 (au)				
					Mean	Intra-assay precision CV% sections	Inter-assay precision CV% experiments	Q90 mean	Ranking
DMD 3	3	1	2	777	255	6%	10%	635	4
	4	17	2	803	241	3%		662	
	7	527	4	1849	208	10%		589	
DMD 1	3	1	2	976	286	8%	14%	701	3
	4	17	2	1075	298	2%		729	
	7	527	4	2103	228	3%		604	
DMD 4	5	142	2	572	306	4%	17%	843	2
	6	324	2	579	427	5%		1152	
	7	527	4	2012	354	2%		997	
DMD 5	5	142	2	583	406	4%	2%	1094	1
	7	527	4	1375	397	6%		1148	

The intra-assay precision (CV%) was calculated for the mean dystrophin intensity of multiple sections of a biopsy/sample analyzed in the same experiment. Inter-assay precision between experiments performed on different days was assessed in two ways by ranking of the samples based on the dystrophin intensity in each experiment and by calculating the CV% from the mean dystrophin intensity in each experiment (different experiments 3, 4, 5, 6, and 7 were performed over the course of 1.5 years with the day of experiment shown in the table).

CV, coefficient of variation; DMD, Duchenne muscular dystrophy.

The reproducibility of dystrophin measurement in DMD samples was determined by comparing the dystrophin intensity of multiple sections of a DMD biopsy within one experiment (intra-assay precision) and between different experiments performed on separate days (inter-assay precision). The mean dystrophin intensity of four sections of the same DMD sample analyzed in the same experiment showed a CV% of between 2 and 10%. In experiments with two sections, CV% ranged from 4 to 10% (Table 4). The inter-assay precision was assessed in three independent experiments performed by different operators on three different days over the course of 1.5 years. Using the dystrophin intensity measured, the DMD samples were ranked and the order was consistent between the three experiments (Table 4). The CV% for the dystrophin intensity between experiments was calculated to range between 2 and 17% for the DMD biopsies. This indicates that, under these imaging conditions, the ranking and comparison of DMD samples is reproducible. A summary of the experiments performed is given (Table 5).

**Table 5:** Summary of experiments: different operators performing the staining, imaging, and Definiens image processing.

Experiment	Intensity of control beads (% laser intensity)	Operators		
		Staining	Image acquisition	Definiens processing
7	2302 (7%)	1, 9	4, 8	8, 9, 4, 10
6	2137 (7%)	1, 3	2, 4	3, 6, 7
5	2076 (7%)	1, 2	3, 4	3, 4
4	1887 (7%)	2, 6	5, 7	1
3	2295 (7%)	5, 6	4, 3	3, 6, 1
2	340 (1%)	3, 5	1, 4	1, 3
1	N/D	3	3, 4	3, 4, 5

N/D, not determined.

### Dystrophin Intensities in Pre- and Post-Treatment DMD Muscle Biopsies

**Figure 5:**
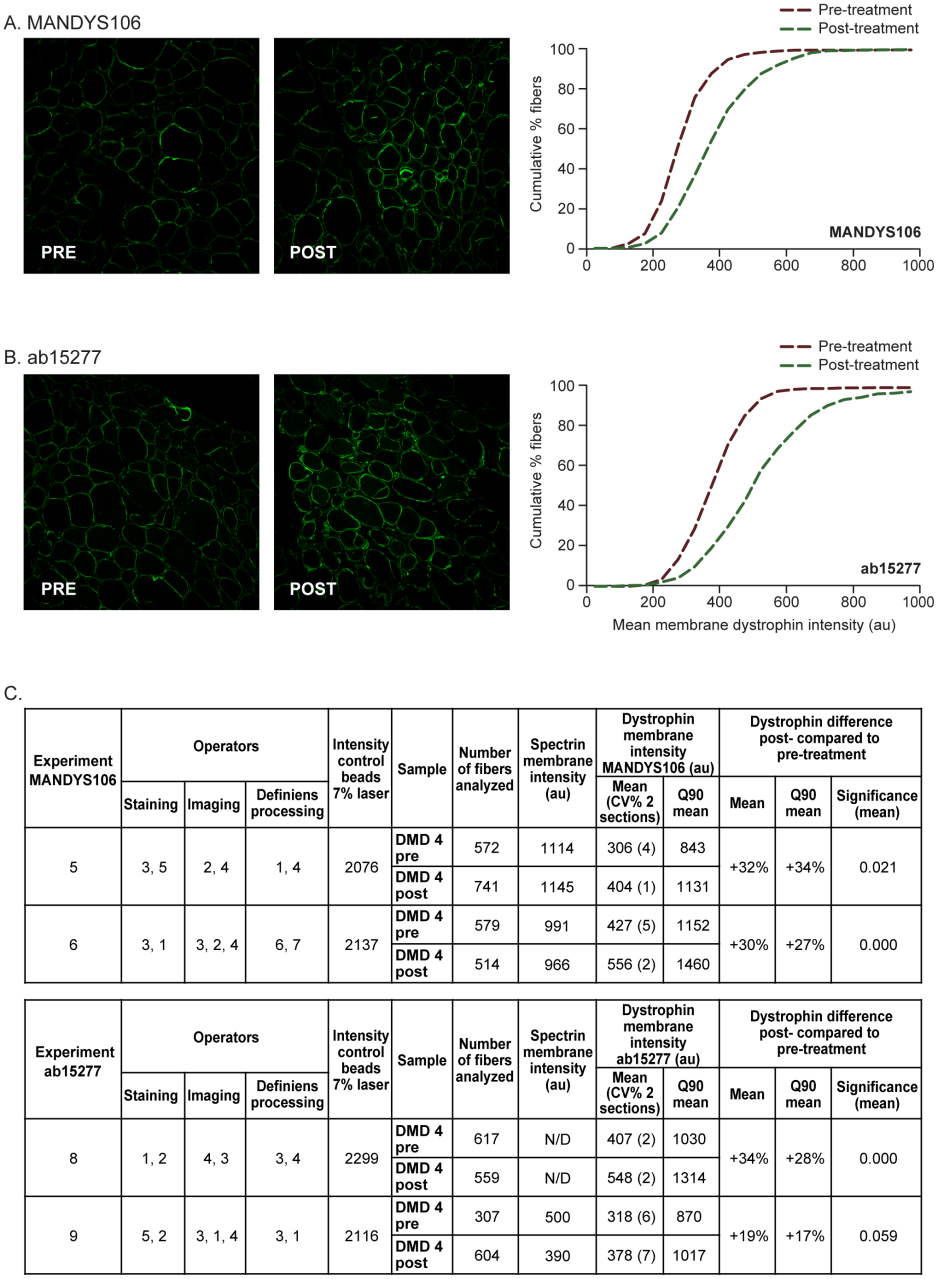
Dystrophin intensities in pre- and post-treatment biopsies from DMD patient 4 (deletion exon 45) using two different anti-dystrophin antibodies. **A.** Representative immunofluorescence images (1 of 10) and dystrophin intensity measurements of the muscle fiber population of pre- and post-treatment biopsies using MANDYS106 (left) and the corresponding cumulative graphs (right). **B.** Representative immunofluorescence images (1 of 10) and dystrophin intensity measurements of the muscle fiber population of pre- and post-treatment biopsies using ab15277 (left) and the corresponding cumulative graphs (right). **C.** Reproducibility of the detection of dystrophin intensity difference between post-treatment and pre-treatment biopsies using MANDYS106 and ab15277. Percentage dystrophin difference  =  ([dystrophin intensity post-treatment – dystrophin intensity pre-treatment]/dystrophin intensity pre-treatment ×100). The statistical significance was calculated using a mixed model analysis taking into account the following covariance parameters: variance between images of one section, variance between sections, variance between slides. The mean difference was significant at the 0.05 level. No difference was observed for the spectrin staining. (au: arbitrary units; CV: coefficient of variation; N/D: not determined).

Using the optimized staining protocol, confocal image acquisition and Definiens analysis procedures, it is possible to accurately, objectively, and reproducibly assess differences in dystrophin levels in DMD between pre- and post-treatment muscle samples from clinical studies in DMD aiming to correct the open-reading frame and restore (internally truncated) dystrophin expression in patients with DMD. As an example, in a patient with DMD with an exon 44 flanking deletion treated for 5 weeks with 12 mg/kg PRO044 (by subcutaneous injections), the average dystrophin mean intensity in the fiber population was measured in the post and pre treatment biopsy. The % difference in dystrophin intensity between the post and pre-treatment biopsy was calculated (% dystrophin difference  =  ([dystrophin intensity post-treatment – dystrophin intensity pre-treatment]/dystrophin intensity pre-treatment ×100). The dystrophin intensity was higher in the post-treatment biopsy by 32% for MANDYS106 and 30% for ab15277, when compared with the pre-treatment biopsy (Figure 5). Also, the cumulative fiber plots for mean dystrophin intensities showed a clear shift to higher intensities for the post-treatment biopsies. Duplicate sections showed an intra-assay precision between 1 and 7%. To demonstrate reproducibility, the experiment with each antibody was repeated in a second experiment and the average membrane dystrophin mean intensity per fiber in the post-treatment biopsy was similarly higher by 30% using MANDYS106 and 19% using ab15277, compared to the pre-treatment biopsy. The difference between the pre- and post-treatment dystrophin levels was statistically significant (*P*<0.05) (Figure 5C). Hence, a difference in dystrophin intensity between two biopsies of the same DMD patient can be reproducibly demonstrated. Whether this is a treatment effect would require analysis of multiple biopsies, including placebo-treated control subjects.

## Discussion

Dystrophin analysis in muscle biopsies is especially relevant for potential therapeutic approaches that aim to increase dystrophin expression in patients with DMD, such as recent efforts in gene replacement, read-through of stop codon mutations, or antisense-induced exon skipping. Measurement of dystrophin expression can provide proof-of-mechanism or support dose selection [8], [14]. The occurrence of trace dystrophin levels in muscle fibers, and in addition the incidence of strong dystrophin-positive revertant fibers in patients with DMD, should be reported and taken into account in the interpretation of dystrophin assessments using tissue homogenates such as RT-PCR, Western blot, or mass-spectrometer analysis. Generally, Western Blot provides information on the total amount of dystrophin present and immunofluorescence gives insight on the functional dystrophin present at the sarcolemma of individual fibers and thus the methods are complementary. IFA of muscle cross-sections can be used to differentiate a potential drug effect from trace and revertant fiber dystrophin expression. IFA can also focus on the dystrophin levels in muscle fibers while avoiding fibrotic areas and fatty cells that increase with disease progression. Nevertheless, comparison of two biopsies remains best when both biopsies have been sampled from areas of similar relative muscle content. A disadvantage for IFA may be that image analysis of cross-sections requires relatively well preserved morphology of the biopsy and to obtain and preserve high quality samples without freeze artifacts may be a challenge in multicenter, international trials.

We present here an objective, sensitive, reproducible, and standardized IFA method to assess dystrophin intensity levels per individual fiber in an entire fiber population in a semi-automated manner. The current IFA method uses double-staining for dystrophin and spectrin and the results have shown that the assay is (i) specific, (ii) sensitive, (iii) reproducible, and (iv) operator-independent. Specificity was shown by comparing the dystrophin-specific antibody staining with isotype staining for aspecific binding and by including a genetic control lacking the dystrophin epitope for the MANDYS106 antibody. Sensitivity was demonstrated by the detection of even minor differences at a wide range of dystrophin signal intensities from high dystrophin expression levels in healthy control samples to intermediate levels for BMD and low levels for DMD samples. Reproducibility was shown by the low intra-assay precision with a CV% between different sections of a biopsy generally less than 10%. The inter-assay precision between separate experiments performed over long interval periods was calculated to have a CV% between 2 and 17% for the DMD samples measured. The ranking of different biopsies and the percentage difference between post- and pre-treatment biopsies remained highly similar between different experimental days and using two different antibodies binding to the rod domain (MANDYS106) or to the C terminus of the dystrophin protein (ab15277). A calibration curve or quality control standards are not available for dystrophin measurement by immunofluorescence and the staining and absolute fluorescence can differ somewhat between experimental days. Therefore, a paired comparison of biopsies provides the best sensitivity and reproducibility when analyzed within the same experiment. Finally, the method employs semi-automated fiber recognition software using the spectrin staining as a mask. Therefore, multiple samples can be assessed relatively quickly and independent of operator bias, which is a major advantage over methods based on visually counting cells or selecting random areas for measurement [4]–[6].

Spectrin was chosen as the membrane control protein to detect individual fibers and to define a mask for measurement of the dystrophin signal intensity, as it shows a better co-localization with dystrophin than laminin (data not shown). In a previous report, spectrin levels were assessed by averaging all fibers in an image, and no statistical difference between disease and healthy muscle samples was found [4]. We observed, however, typically higher spectrin intensities in the BMD and DMD samples than in control samples. This is in agreement with earlier reports describing qualitative assessments of immunofluorescence images for spectrin in diseased tissue from neuromuscular diseases including DMD [12] and in avian dystrophic muscle [13]. In the current method, spectrin is used only as a membrane identifier, not for normalization of the dystrophin signal, so any such potential disease pattern would not affect the outcome. We are currently gathering data to assess trends in spectrin and dystrophin in a large number of DMD samples.

Employing a method that takes individual fibers into account and uses software for semi-automated measurements to reduce operator bias appears especially important in DMD because baseline biopsy samples show levels of trace dystrophin in almost all fibers. Therefore, the distribution of dystrophin expression over the different muscle fibers and the positive shift to a higher dystrophin (staining) intensity for the entire fiber population become key differentiators to detect a dystrophin difference upon treatment. Interestingly, the method showed that dystrophin intensities vary widely between fibers within one biopsy, and two- to threefold between fibers with the lowest and highest mean dystrophin intensity per fiber. Also, differences in the average dystrophin intensity were observed between different muscle groups and between different donors, which is in agreement with up to 40% difference between 2 controls reported previously using both epifluorescence and confocal microscopy [4]–[6]. The difference in dystrophin expression between fibers, muscle groups, and donors may be correlated to transcript levels, but it may also be regulated post-transcriptionally by, for instance, non-coding RNAs. In addition to the lack of a standardized control, dilutions are not possible and, hence, dilution linearity and accuracy cannot be demonstrated by immunofluorescence analysis. Various recent studies, that have depicted dystrophin intensity as % of control by immunofluorescence analysis, have reported a wide variety of trace dystrophin levels for DMD patients between 0% and 25% when compared to healthy control dystrophin intensity and those extrapolated intensity levels also differed dependending on the antibody used [4], [5], [15], [16]. The wide variability in results and dependency on antibody indicate that proportional extrapolation from control is likely inaccurate. Although Western Blot analysis is generally less sensitive compared to immunofluorescence analysis, it does allow for direct comparison with different dilutions of control sample and thereby improved accuracy. Taylor et al. (2012) [4] and Anthony et al. (2014) [15] detected higher % of control levels of dystrophin for immunofluorescence compared to Western blot analysis for the same DMD sample. All these points are highly relevant for clinical studies aiming to assess dystrophin levels in pre- and post-treatment muscle biopsies or express dystrophin levels in percentages of control. The choice of the muscle to obtain a biopsy, the consistent use of that muscle during a study for that patient, and, in case of normalization to control, the choice of the healthy donor tissue may directly affect the outcome. It is, therefore, difficult to compare results between studies that have used different muscles and different reference muscle samples.

Hence, the presented immunofluorescence analysis provides in general high sensitivity and localization of dystrophin expression and in this study we have presented a methodology that provides a reproducible result with high precision when comparing biopsies. However, the absolute difference between two biopsies should be interpreted with care. In the presented example, a reproducible difference of 30% in dystrophin intensity has been measured, underlining that the assay can reliably detect small differences. In the DMD pre- and post-treatment biopsies, the dystrophin levels were quite low. Using Western blot analysis, biopsies for non-treated DMD patients with 44 flanking deletions showed levels in the range of 1–10% of control (data not shown). Interpretation of the result is beyond the scope of this report and more patients should be evaluated and compared to placebo treated patients to establish whether the difference is a change related to treatment. Furthermore, comparison of such result with clinical performance would be needed to establish whether a small difference of 30% from baseline would be sufficient or whether 2 or more fold change would be needed for clinical benefit.

In conclusion, we have developed a semi-automated, objective, and reproducible immunofluorescence method optimized for assessing dystrophin levels in muscle biopsies from BMD or DMD patients in natural history studies or clinical studies with compounds aiming to restore dystrophin expression. It is highly sensitive for small differences between samples with an intra-assay precision with a CV% lower than 10%, allowing careful relative-quantitative comparison of biopsies within one experiment. Such comparison of a pre- and post-treatment biopsy within one experiment was also shown to be reproducible on different experimental days and independent of operators. This method has been tested as part of an international consortium initiative, the Biochemical Outcome Measures study group, focusing on the optimization of dystrophin assays and the standardization between different laboratories and operators; the results of this initiative will be reported in a forthcoming publication by the study group. Using the assay, variability was noted between donors of (non-DMD) control samples and, also, different muscle groups appear to exhibit different expression levels. Therefore, the choice and consistent use of muscle samples should be taken into account in clinical studies when analyzing biopsies for dystrophin expression and interpreting the results.

## Supporting Information

Figure S1 **A. Representative immunofluorescence images (1 of 10 images) from the tibialis anterior muscle samples (from one control subject (CTRL2), two patients with DMD and four patients with BMD.** Analysis was performed in the same experiment using the antibody ab15277 and ‘control’ imaging settings (1% laser intensity). **B.** Corresponding dystrophin membrane intensity distribution in the fiber populations analyzed (cumulative graphs). (au: arbitrary units; BMD: Becker muscular dystrophy; CTRL: control; DMD: Duchenne muscular dystrophy).
(TIF)

Figure S2 **Spectrin and dystrophin intensity in the tibialis anterior muscle from control and pre-treatment DMD and BMD muscle samples assessed by immunofluorescence staining and Definiens analysis.** In general, spectrin levels appear somewhat lower in control samples but no consistent differences between DMD and BMD samples were observed. **A.** Tibialis anterior muscle from healthy control 2 was compared with two patients with DMD with exon 51 flanking deletions (DMD 1: deletion exon 48–50, DMD 2: deletion exon 45–50) and four patients with BMD); Dystrophin levels (from a MANDYS106-double staining with an anti-spectrin antibody) are different between the DMD, BMD and control samples. **B.** Spectrin levels for these same samples, measured from the staining with isotype for MANDYS106 with an anti-spectrin antibody. **C.** Tibialis anterior muscle from healthy control 1 was compared with two patients with DMD with exon 51 flanking deletions (DMD 1: deletion exon 48–50 and DMD 2: deletion exon 45–50). **D.** Tibialis anterior muscle from healthy control 2 was compared with two patients with DMD with exon 44 flanking deletions (DMD 4: deletion exon 45; DMD 5: deletion exon 45) (au: arbitrary units; BMD: Becker muscular dystrophy; CTRL: control; DMD: Duchenne muscular dystrophy).
(TIF)

Table S1 **Overview DMD114117 and PRO044 hospital committees.**
(DOCX)
