# Supplementary figures and images for: A Sensitive, Reproducible and Objective Immunofluorescence Analysis Method of Dystrophin in Individual Fibers in Samples from Patients with Duchenne Muscular Dystrophy
Source: PLoS One. 2014 Sep 22;9(9):e107494. doi: 10.1371/journal.pone.0107494 (PMC4171506; doi:10.1371/journal.pone.0107494)

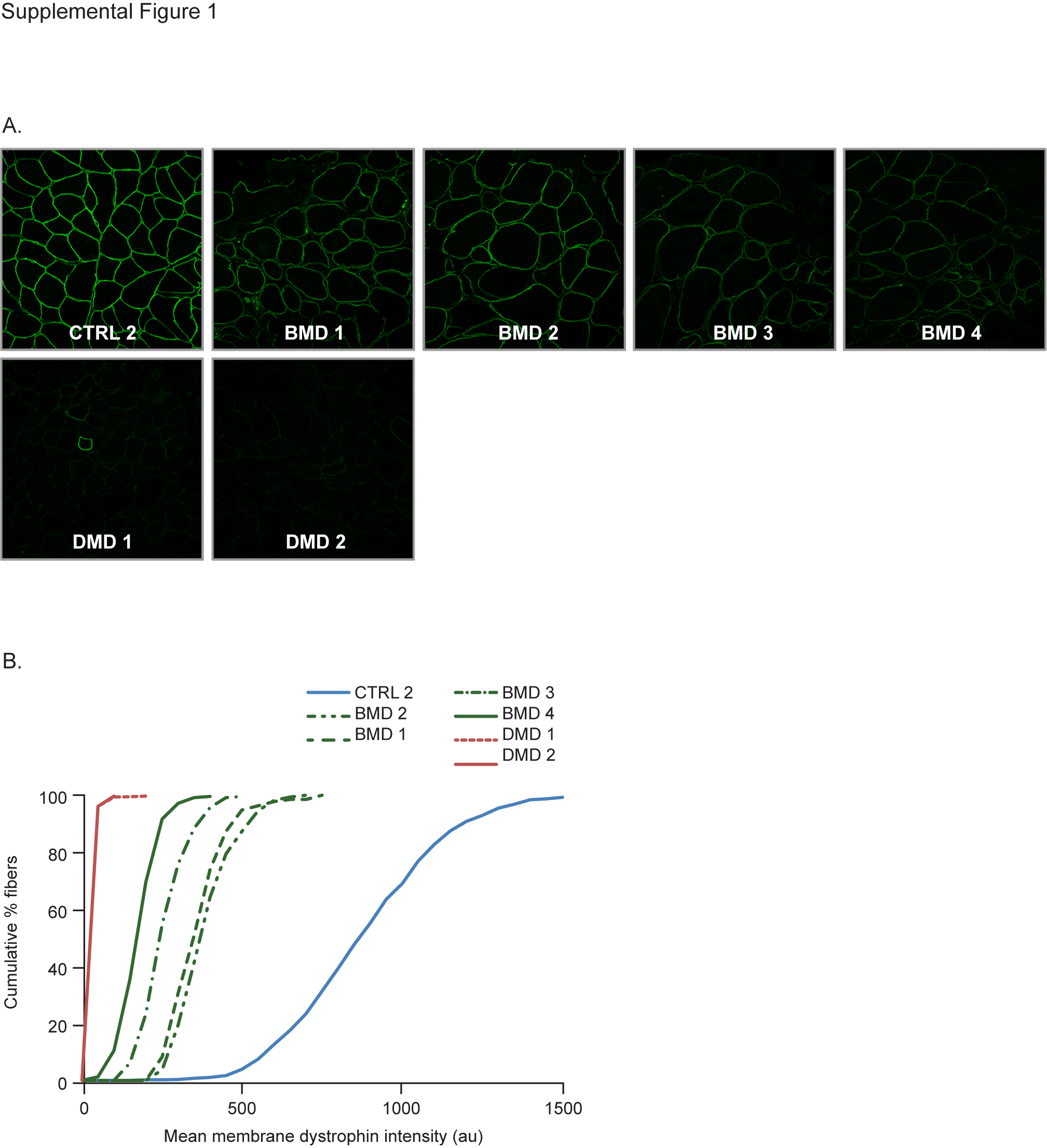

Supplement: Figure S1 — A. Representative immunofluorescence images (1 of 10 images) from the tibialis anterior muscle samples (from one control subject (CTRL2), two patients with DMD and four patients with BMD. Analysis was performed in the same experiment using the antibody ab15277 and ‘control’ imaging settings (1% laser intensity). B. Corresponding dystrophin membrane intensity distribution in the fiber populations analyzed (cumulative graphs). (au: arbitrary units; BMD: Becker muscular dystrophy; CTRL: control; DMD: Duchenne muscular dystrophy). (TIF) [file pone.0107494.s001.tif]

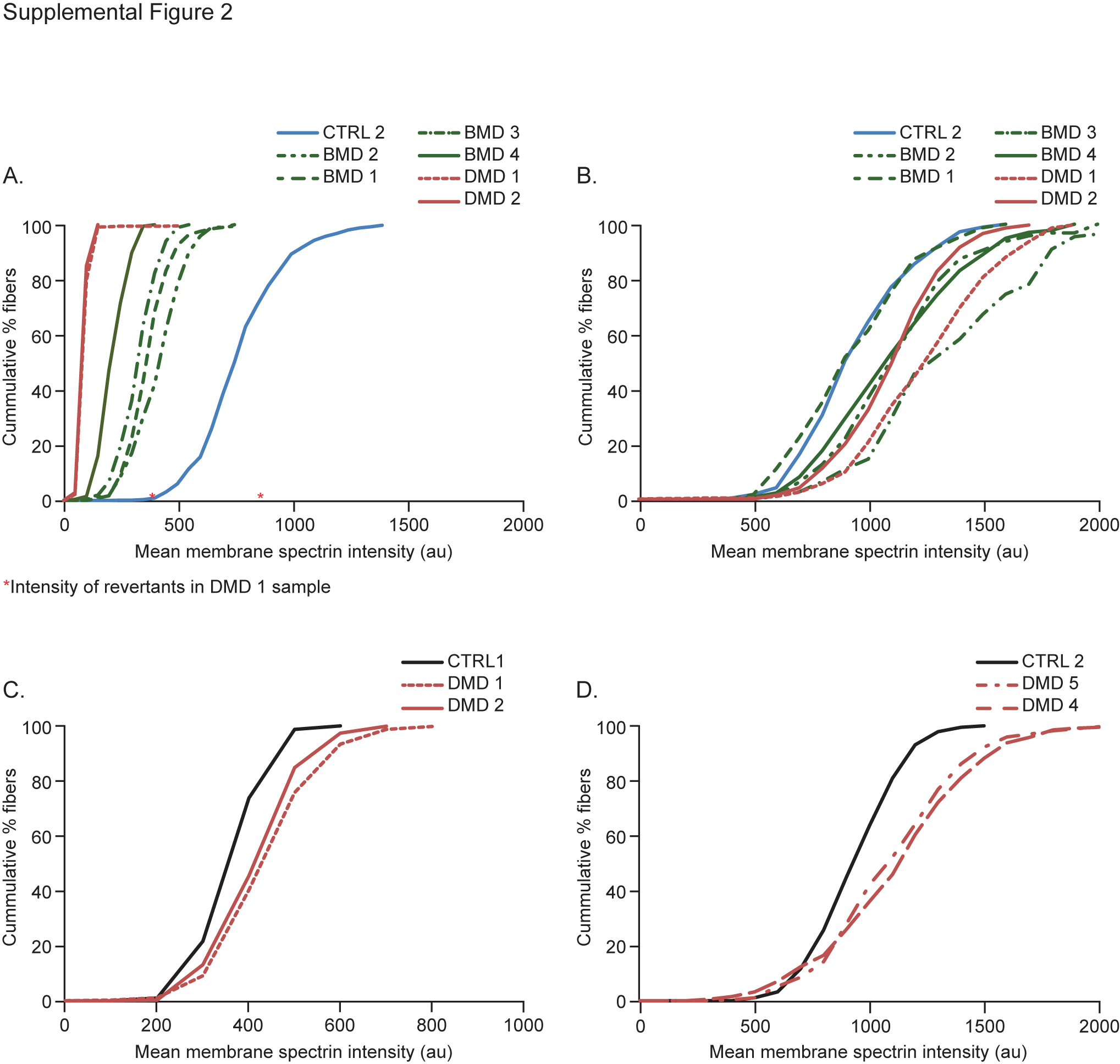

Supplement: Figure S2 — Spectrin and dystrophin intensity in the tibialis anterior muscle from control and pre-treatment DMD and BMD muscle samples assessed by immunofluorescence staining and Definiens analysis. In general, spectrin levels appear somewhat lower in control samples but no consistent differences between DMD and BMD samples were observed. A. Tibialis anterior muscle from healthy control 2 was compared with two patients with DMD with exon 51 flanking deletions (DMD 1: deletion exon 48–50, DMD 2: deletion exon 45–50) and four patients with BMD); Dystrophin levels (from a MANDYS106-double staining with an anti-spectrin antibody) are different between the DMD, BMD and control samples. B. Spectrin levels for these same samples, measured from the staining with isotype for MANDYS106 with an anti-spectrin antibody. C. Tibialis anterior muscle from healthy control 1 was compared with two patients with DMD with exon 51 flanking deletions (DMD 1: deletion exon 48–50 and DMD 2: deletion exon 45–50). D. Tibialis anterior muscle from healthy control 2 was compared with two patients with DMD with exon 44 flanking deletions (DMD 4: deletion exon 45; DMD 5: deletion exon 45) (au: arbitrary units; BMD: Becker muscular dystrophy; CTRL: control; DMD: Duchenne muscular dystrophy). (TIF) [file pone.0107494.s002.tif]
