# Supplementary material for: A Sensitive, Reproducible and Objective Immunofluorescence Analysis Method of Dystrophin in Individual Fibers in Samples from Patients with Duchenne Muscular Dystrophy
Source: PLoS One. 2014 Sep 22;9(9):e107494. doi: 10.1371/journal.pone.0107494 (PMC4171506; doi:10.1371/journal.pone.0107494)
Supplement: Table S1 — Overview DMD114117 and PRO044 hospital committees. (DOCX) [file pone.0107494.s003.docx]

| **DMD114117 participating sites** | | | | |  |
| --- | --- | --- | --- | --- | --- |
| **Country** | | **City** | | **Ethics committee of hospital** |  |
| Germany | | Freiburg | | Universitätsklinikum Freiburg (Kinderklinik) |  |
| Germany | | Essen | | Uniklinik Essen |  |
| Netherlands | | Nijmegen | | Radboud University Nijmegen |  |
| UK | | Newcastle | | Dental Hospital |  |
| Belgium | | Gent | | UZ Gent |  |
| UK | | London | | National Hospital For Neurology and Neurosurgery |  |
| France | | Paris | | University Pierre et Marie Curie |  |
| Australia | | Westmead | | The Children's Hospital at Westmead |  |
| Australia | | Parkville | | The Royal Children's Hospital |  |
| Spain | | Barcelona | | Hospital Sant Joan de Déu Barcelona |  |
| Spain | | Valencia | | Hospital Universitario La Fe |  |
| Turkey | | Ankara | | Ankara Numune Teaching and Research Hospital |  |
| Israel | | Jerusalem | | Hadassah-Hebrew University Medical Center |  |
|  |  | |  |  |  |
| **PRO044-CLIN01 participating sites** | | | | |  |
| **Country** | | **City** | | **Ethics committee of hospital** |  |
| Italy | | Ferrara | | Universita di Ferrara (Sezione di Genetica Medica) (=Ospedale Sant Anna) |  |
| Netherlands | | Leiden | | Leiden University Medical Center |  |
| Sweden | | Göteborg | | The Queen Silvia Children’s Hospital (=Drottning Silvias barn-och ungdomssjukhus) |  |
| Belgium | | Leuven | | UZ GASTHUISBERG Leuven |  |
